# Safety of CDK4/6 Inhibitors Combined with Radiotherapy in Patients with Metastatic Breast Cancer: A Review of the Literature

**DOI:** 10.3390/curroncol30060415

**Published:** 2023-06-06

**Authors:** Rejane Franco, Jeffrey Q. Cao, Michael Yassa, Tarek Hijal

**Affiliations:** 1 Division of Radiation Oncology, McGill University Health Centre, Montreal, QC H4A 3J1, Canada; 2 Division of Radiation Oncology, Department of Oncology, University of Calgary, Calgary, AB T2N 4N2, Canada; jeffrey.cao@albertahealthservices.ca; 3 Division of Radiation Oncology, Hôpital Maisonneuve-Rosemont, CIUSSS de l’Est-de-l’Île-de-Montréal, Montreal, QC H1T 2M4, Canada; myassa.hmr@ssss.gouv.qc.ca

**Keywords:** cyclin-dependent kinase inhibitors, radiotherapy, breast cancer

## Abstract

Recent evidence suggests that cyclin-dependent kinase 4/6 (CDK4/6) inhibitors significantly improve progression-free survival and overall survival among metastatic breast cancer patients. However, given the effects on cell cycle arrest, there is potential for CDK4/6 inhibitors and radiotherapy (RT) to work synergistically, enhancing the effect and toxicities of RT. A comprehensive review of the literature on the combination of RT and CDK4/6 inhibitors was performed with 19 eligible studies included in the final analysis. A total of 373 patients who received radiotherapy combined with CDK4/6 inhibitors were evaluated across 9 retrospective studies, 4 case reports, 3 case series, and 3 letters to the editor. The CDK4/6 inhibitor used, RT target, and RT technique were assessed in terms of toxicities. This literature review demonstrates generally limited toxicities with the combination of CDK4/6 inhibitors and palliative radiotherapy to metastatic breast cancer patients. The current evidence is nonetheless limited, and further results of ongoing prospective clinical trials will help clarify whether these treatments can be safely combined.

## 1. Introduction

Multiple treatment options are available for patients with hormone-receptor-positive metastatic breast cancer, such as estrogen receptor antagonists and aromatase inhibitors, which can be used alone or in combination with other systemic treatments to improve outcomes. More recently, the association of these agents with cyclin-dependent kinase 4/6 (CDK4/6) inhibitors has demonstrated substantial improvement in progression-free survival [1,2] and overall survival [3,4], additionally supporting the standard use of CDK4/6 inhibitors and antiestrogen therapy combination. To date, the FDA and the European Medicines Agency (EMA) have approved palbociclib, ribociclib, and abemaciclib for use in localized or metastatic breast cancer after the publication of several positive trials, including PALOMA, MONALEESA, and MONARCH [5,6,7], respectively.

This class of agents acts through the blockage of cyclin-dependent kinases 4 and 6 and the inhibition of the tumor suppressor function of the retinoblastoma (Rb) protein, arresting the cell cycle at the G1 to S phase restriction point and causing senescence of the malignant cells [8]. Given the effects on cell cycle arrest, there is potential for CDK4/6 inhibitors and radiotherapy (RT) to work synergistically, enhancing the effect of RT due to cancer cells’ arrest in a more radiosensitive phase [9].

In vitro, an increase in apoptosis and inhibition of colony formation were observed when palbociclib was combined with RT in glioblastoma-patient-derived cells [10]. Non-small-cell lung cancer with functional p53 and RB proteins had enhanced sensitivity to radiotherapy after abemaciclib regardless of RAS or EGFR status [11]. Moreover, treatment with palbociclib before and during 5 daily fractions of subtotal body irradiation in murine models exacerbated gastrointestinal acute radiation syndrome [12].

RT is an integral part of multidisciplinary treatment for breast cancer at all stages, and up to 50% of patients with metastatic breast cancer will require palliative RT during their disease course [13]. Inhibitors of CDK4/6 have been widely adopted, although numerous questions regarding their toxicity and safety remain unanswered. There are currently no large-scale or phase 3 data regarding the safety and efficacy of the combination of CDK4/6 inhibitors with RT. In phase 3 trials used to approve CDK4/6 inhibitors, the drug administration was held during palliative RT in patients with symptomatic metastases, and there was no specific analysis of toxicity performed in patients who had received palliative radiotherapy.

The purpose of this paper is to review the literature on the combined use of RT and CDK4/6 inhibitors and evaluate their toxicity in hormone-receptor-positive metastatic breast cancer.

## 2. Materials and Methods

**Figure 1:**
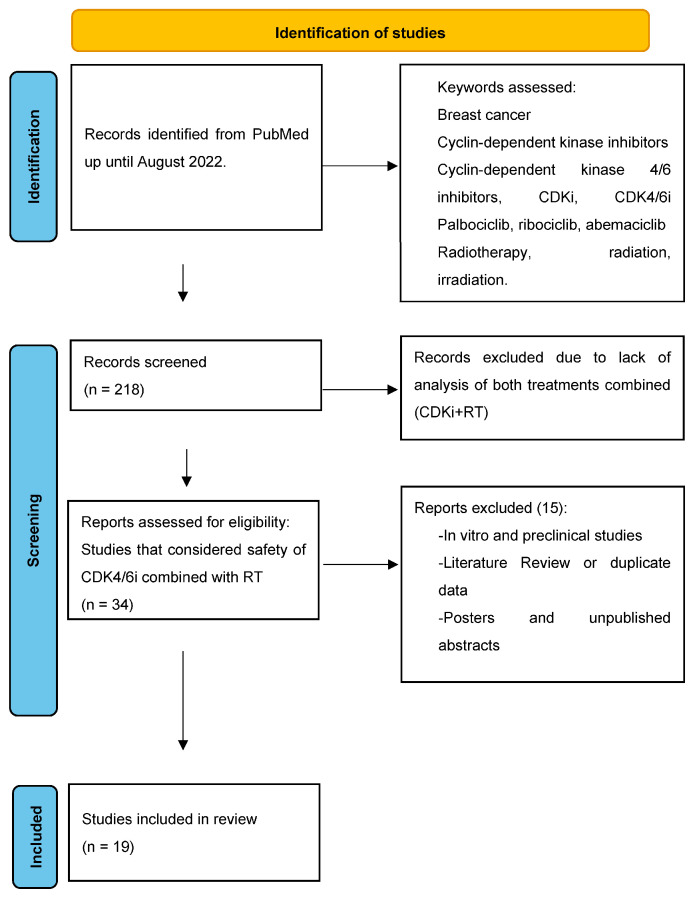
Identification of studies.

A comprehensive review of the literature up until August 2022 was performed by searching in the PubMed database a combination of the following keywords: breast cancer, cyclin-dependent kinase inhibitors, cyclin-dependent kinase 4/6 inhibitors, CDKi, CDK4/6i, palbociclib, ribociclib, abemaciclib, radiotherapy, radiation, and irradiation. Two hundred and eighteen articles were identified. The abstracts of these articles were reviewed, and 34 articles fit the search criteria of clinical studies assessing the effect of combining radiotherapy and CDK4/6is. The references of these 34 articles were reviewed to find additional papers that might have been missed by the original search. Finally, the full texts of these relevant articles were evaluated to assess eligibility. The exclusion criteria were in vitro and preclinical studies, literature review that evaluated publications considered in our analysis, and posters/abstracts that had not been published. A total of 19 eligible studies were included in the final analysis, as shown in Figure 1.

Data on each study were gathered as follows: name of the principal author, year of publication, study design, number of patients available for analysis, median age at RT treatment, number of RT courses, RT technique, RT dose/fractionation, RT target, CDK4/6 inhibitor used, hormonal therapy given at the time of RT, timing of RT in relation to CDK4/6i, toxicities, and individual patient data when available.

## 3. Results

Based on the analysis of the 19 selected articles, 373 patients who received radiotherapy combined with CDK4/6 inhibitors were evaluated across 9 retrospective studies [13,14,15,16,17,18,19,20,21], 4 case reports [22,23,24,25], 3 case series [26,27,28], and 3 letters to the editor [29,30,31], as shown in Table 1.

Palbociclib was notably the most prescribed drug, followed by ribociclib and abemaciclib. There were 10 publications [17,18,19,22,24,25,26,28,29,30] describing patients who received exclusively palbociclib and 7 publications [13,14,15,16,20,21,31] that included the three drugs, but of these; palbociclib was also the most prevalent administered drug. In exceptions to this, Meattini [27] and Erjan [23] presented data on patients who received only ribociclib, with the first describing five cases and the second reporting a case.

Figura et al. [14] examined the effects of the combined treatment restricted to patients diagnosed with brain metastases. All the remaining authors described different target sites altogether, bone metastasis being the most commonly irradiated target, accounting for about 90.8% of the cases. The spine was the most frequently irradiated bone, generally with no distinction between cervical, thoracic, and lumbar regions, followed by pelvic bones. Brain, visceral metastases, nodes, and chest wall were also among the metastasis targets reported, representing, respectively, 6.2%, 1.2%, 1.4%, and 0.4% of the cases. Alongside radiotherapy for metastatic lesions, Beddock et al. [18] and Al-Rashdan et al. [21] also described together the results of 48 patients receiving locoregional breast radiotherapy.

Typically, patients received more than one course of radiation, and the prescribed RT doses were palliative, ranging from 8 to 37.5 Gy with exception to supraclavicular nodes being treated with 60 Gy [15,22] and locoregional breast radiation receiving around 50.4 Gy [18].

With regard to the radiation technique, Figura et al. [14], with a restricted group of brain metastasis patients, was the unique publication, which adopted only one radiation technique—SBRT/FSRT. The remaining authors, when providing data, generally reported patients receiving 3D conformal RT among a range of techniques.

Authors assessed toxicities by most commonly using the National Cancer Institute Common Terminology Criteria for Adverse Events (CTCAE), versions 4.0 or 5.0. However, not every publication, in particular some case reports and case series [24,25,26,27,31], described the method by which the toxicities were measured.

Hematological toxicities were the most prevalent side effect, with neutropenia and leukopenia affecting over 43% and 29% of patients, respectively, in publications assessing hematological side effects, followed by anemia and thrombocytopenia. All grade 4 hematological toxicities presented as neutropenia, as reported by Ippolito [15], Guerini [16], Ratosa [20], and Meattini [27]; these are shown in Table 2.

Aside from hematologic toxicity, dermatitis was the second most common side effect, accounting for close to 7% of all patients, with mainly grade 2 severity in 58% of the cases, although there were nine cases of grade 3 severity (37,5%). Diarrhea was seen in 20 patients (5,8%), including two grade 4 cases that underwent hip irradiation in conjunction with ribociclib, and were reported by Meattini et al. [27] and Al-Rashdan et al. [21], as shown in Table 3.

Colitis was reported in four patients, with two cases reported by Dasgputa et al. [24] and Kawamoto et al. [30] and the others documented by van Aken et al. [28] and Guerini et al. [16]. All colitis cases were classified as having grade 3 toxicity, in the context of palbociclib given in conjunction with 3D conformal RT to pelvic bones. Kawamoto et al. [30] noted that a preradiation palbociclib treatment had already resulted in grade 1 diarrhea in a patient who developed radiation-induced colitis.

Fatigue and nausea accounted for a substantial proportion of cases in the retrospective study by Chowdhary et al. [17] involving 31% and 25% of patients, respectively, all grade 2. Meanwhile, none of the remaining authors noted fatigue or nausea as a side effect.

Some other reported toxicities with lower prevalence were edema, headache, constipation, mucositis, and gastritis.

There were also two radiation recall cases reported by Erjan et al. [23] and David et al. [26]. In the first case, a grade 2 chest wall dermatitis developed four weeks after radiation therapy, while David et al. [26] reported a case of grade 5 pneumonitis resulting in patient death after palbociclib administration. To the latter, radiotherapy was administered 4 months before the drug prescription at a dose of 20 Gy in 5 fractions to mediastinal nodes. In this case, radiation recall pneumonitis was the most likely cause of death, although the patient had also received a mechanistic target of rapamycin inhibitor, raising speculation as to whether it was caused by RT and CDK4/6i.

## 4. Discussion

It is still unknown what mechanisms underlie the combined effects of CDK4/6 inhibitors and radiation therapy. CDK4/6 inhibitors have radiosensitized cancer cells in preclinical studies by arresting cell cycle, reducing DNA damage repair, enhancing apoptosis, and causing cellular senescence [32]. Since radiosensitizing effects might potentially also affect healthy tissues and lead to toxicity exacerbation, the lack of a substantial body of clinical data might discourage clinicians from prescribing concomitant treatment with RT and CDK4/6 inhibitors, with unnecessary interruption of systemic treatment or even abstention from the benefits of RT. Therefore, here arises the importance of evaluating toxicity rates for combined treatment, while considering the intrinsic toxicity of CDK4/6 inhibitors when administered alone.

It is common for CDK4/6 inhibitors to cause myelosuppression, especially neutropenia. The underlying mechanism is believed to involve cytostatic activity in the bone marrow, since cell cycle arrest without apoptosis has been demonstrated in vitro, which is also consistent with low rates of febrile neutropenia across all CDK4/6 inhibitor trials [33]. Hematologic adverse events are influenced by CDK6 targeting, since CDK6 is more prominent in the bone marrow, where it regulates hematopoietic and leukemic stem cell transcription [34]. Consequently, palbociclib and ribociclib are more likely to cause hematologic toxicity than abemaciclib, which strongly targets CDK4 [35].

Across the PALOMA and MONALEESA clinical trials, all grade neutropenia occurred with palbociclib and ribociclib in 80% and 69% of patients, respectively, and leukopenia affected 50% and 28% of patients [36,37]. The PALOMA-2 trial [38], when exploring the incidence of hematological adverse effects in patients treated with CDK4/6 inhibitors, reported neutropenia grades 3 and 4 in up to 66% of patients, a number that is not far from the 43% observed amid the studies included in this literature review, especially considering that blood counts are seldom collected during short palliative radiation treatments. Dasgputa et al. [24] found neutropenia as the most common grade 3 or 4 adverse event when used along with letrozole or fulvestrant, accounting for 50%–65% of patients, although febrile neutropenia was rare. Chowdhary et al. [17] observed grade 1 neutropenia in 31.3% of patients before RT, with only one additional patient developing G2 neutropenia after RT, raising the rate to 37.6%.

Ippolito et al. [15] evaluated the safety of palliative RT and concomitant palbociclib or ribociclib treatment in 16 patients. Those patients who developed grade 3 neutropenia (43.7%) from previous CDK4/6i cycles did not develop worsening neutropenia after palliative RT, and according to the authors, radiotherapy does not seem to exert an additional myelosuppressive effect.

Even though all three CDK4/6 inhibitors are likely to have similar efficacy, their toxicity profiles are different. It is likely that abemaciclib’s superior affinity for CDK4 over CDK6 led to its possible dose-limiting toxicity of diarrhea, whereas palbociclib and ribociclib are associated with neutropenia. Patients who received palbociclib had grade 1 or 2 diarrhea incidence of 19.1%, but no higher grade. Conversely, the most frequent side effect with abemaciclib was diarrhea, particularly grade 3 or 4 diarrhea (up to 19.7% versus 4% with palbociclib), which resulted in dose reductions in 30% of patients [39].

Despite gastrointestinal epithelium, of the normal tissues, being one of the most susceptible to antiproliferation CDK4/6 inhibitors’ action, most toxicities reported were fully reversible through dose reduction and supportive care even in grade 3 colitis and diarrhea, with low hospitalization rates.

Kawamoto et al. [30] described a case of irradiation of the iliac bone along part of the bowel, resulting in diarrhea and acute radiation-induced enteritis. It is noteworthy that the patient reported had already developed grade 1 diarrhea after the start of treatment with palbociclib before radiotherapy, and her symptoms had abated with conservative treatment within 3 weeks.

Similarly, Dasgupta et al. [24] reported a 77-year-old woman who developed severe pancolitis following 30 Gy in 10 fractions of radiotherapy to the pelvis and femur combined with palbociclib.

Guerini et al. [16] retrospectively reviewed the records of 18 patients, who all developed limited nonhematological acute toxicity, mainly grade 1, with no need for CDK4/6 inhibitors’ suspension or dose reduction. There was also one case of grade 3 ileitis after the treatment of a bulky bone metastasis involving the L5 vertebra, sacrum, and right ischium in 10 fractions of 3 Gy, which was resolved after conservative management with antibiotics and anti-inflammatory drugs.

In terms of biologically relevant dose (EQD 2 Gy/fr 32.5 Gy), the dose described falls well below the normal bowel radiation tolerance dose of 45–50 Gy in 2 Gy fractions [40]. Since palliative radiotherapy and palbociclib as stand-alone treatments are very unlikely to cause severe gastrointestinal side effects [1], palbociclib might, therefore, have contributed in this case to radiosensitization on normal intestinal tissue. Conceivably, patients who have already had GI toxicity on CDK4/6 inhibitor treatment could be more sensitive to the addition of RT, and conformal radiation techniques may be an effective means of minimizing the potential toxicity of bowel radiation by tailoring the field to these patients.

There may be concerns about synergistic hematological and gastrointestinal toxicity when RT is delivered to pelvic bones and sacrum concurrently with CDK4/6 inhibitors, as these targets account for approximately 35% of active bone marrow in adults [41] and include a significant amount of bowel. Furthermore, patients undergoing palliative radiotherapy with lower baseline white blood cell or neutrophil counts may be at greater risk for acute or late hematologic complications [42,43].

Generally, it is recommended that high conformal treatment techniques, such as IMRT, be used to maintain the dose to the gastrointestinal mucosa at a minimum, especially in patients with large planning target volumes (PTVs) located in the abdominal and pelvic regions. Despite this, Ratosa et al. [20] observed no significant differences in the dosimetric parameters of organs at risk as a result of either combined treatment or temporary discontinuation of CDK4/6i during radiation treatment. RT-treatment-related factors, including PTV, total RT dose prescribed, daily RT dose, and RT site (axial vs. pelvic) or RT technique (conformal vs. nonconformal), did not appear to be significantly related to adverse events, according to the authors.

Clinical trials that have assessed CDK4/6 inhibitors report fatigue and nausea to be among their principal side effects: PALOMA (39% fatigue, 32% nausea), MONALEESA (31% fatigue, 45% nausea), and MONARCH (40% fatigue, 43% nausea). Chowdhary et al. [17] described fatigue and nausea as side effects with significant proportions in their retrospective study, but none of the other studies analyzed in this review mentioned important amounts of these adverse effects.

In view of the evidence pointing to a generally good response when combining RT with CDK4/6i, the concern as to why some patients experience severe adverse effects persists. An experimental Spanish study using palbociclib on lung, colorectal, and breast cancer cells [44] indicates that wild-type p53 is required for palbociclib to function as a radiosensitizer. Palbociclib, on the other hand, loses any radiosensitizing effectiveness when p53 is functionally blocked, but reacquires it once p53 is restored. Ultimately, these data suggest a more patient-tailored treatment, where the combined treatment of CDKI + RT could be used in patients with a nonfunctional p53 pathway, to reduce the risk of toxicity.

The authors assume that the retrospective design of the publications analyzed in this paper imposes limitations to this literature review, including the possibility of missing data, such as adverse events not documented, especially abnormal laboratory tests, since blood counts are not normally collected during radiotherapy, especially during short treatment courses. Further, most publications included a small number of patients, considering in essence the patients who presented considerable side effects. The absence of extended follow-up also did not allow an in-depth analysis of long-term toxicities or response rates, and moreover, the three different CDK4/6 inhibitors studied in a mixed study population of breast cancer patients, as shown by most authors, restrained our conclusions.

Patients with hormone-receptor-positive metastatic breast cancer generally have long survival, particularly if the disease is confined to the bone alone, for which the median survival may exceed 5 years. Furthermore, by administering CDK4/6 inhibitors, the median progression-free survival (PFS) almost doubles when compared with hormonal therapy alone [24]. Besides, there is emerging evidence that ablative RT might improve survival rates for patients with oligometastatic disease [45]. This reflects the majority of data describing patients living longer without visceral metastases, but rather presenting with bone disease. In that context, the consequences of both expected and unexpected long-term side effects cannot be minimized.

We wait for ongoing phase 2 trials, such as the ASPIRE trial (NCT03691493), the PALATINE trial (NCT03870919), and the CLEAR trial (NCT03750396), which will provide more information on the safety of metastasis-directed radiotherapy when combined with concurrent CDK4/6 inhibitors and endocrine therapy.

## 5. Conclusions

In conclusion, our literature review demonstrates overall limited toxicities with the combination of palliative radiotherapy with CDK4/6 inhibitors in patients with metastatic breast cancer. The data are, however, of generally low quality, limited, and subject to multiple biases, as they are extracted from case reports, case series, and retrospective reviews.

Prospective clinical trials are thus needed to better assess the safety of CDK4/6 inhibitors combined with RT. These are currently underway and will further clarify whether these two treatments are safe when combined. In the meantime, clinicians must tread carefully when combining these two treatments to avoid any unexpected toxicity.

## Figures and Tables

**Table 1:** Study baseline.

AUTHOR	STUDY DESIGN	PATIENTS (N)	CDKI4/6 AGENT	CDKI4/6 COMBINED WITH	RT SITE	TECHNIQUE
Kim et al., 2021 [13]	Retrospective analysis	30	Palbociclib (34)	Fulvestrant (13)	Brain (5)	3D-CRT (29)
			Abemaciclib (2)	AI (12)	Bone-spine (19)	IMRT (2)
				TMX (3)	Bone-pelvis (9)	VMAT (4)
				Alone (8)	Bone-other (6)	SBRT (7)
					Other (4)	Electron (1)
David S et al., 2020 [26]	Case series	5	Palbociclib (5)	AI (5)	Mediastinal nodes (1)	N/A
					Right breast (1)	
					Bone-spine (3)	
Hans et al., 2018 [29]	Letter to the editor	5	Palbociclib (5)	Fulvestrant	Bone-pelvis (1)	
					Bone-spine (2)	
					Bone-scapula/humerus (1)	Unknown (4)
					Liver (1)	SBRT (1)
Kawamoto et al., 2019 [30]	Letter to the editor	1	Palbociclib	Fulvestrant	Bone-pelvis	3D-CRT
Figura et al., 2019 [14]	Retrospective analysis	15	Palbociclib (10)	AI (16)	Brain (15)	SBRT (26)
			Abemaciclib (5)	Fulvestrant (20)		FSRT (16)
Ippolito et al., 2019 [15]	Retrospective analysis	16	Palbociclib (13)	N/A	Bone-pelvis (6)	3D-CRT (19)
			Ribociclib (3)		Bone-spine (4)	IMRT (2)
					Bone-others (4)	VMAT (3)
					Chest wall-skin (1)	
					Nodal (1)	
Guerini et al., 2020 [16]	Retrospective analysis	18	Palbociclib (9)	Fulvestrant (10)	Bone-spine (11)	3D-CRT (29)
			Ribociclib (6)	AI (8)	Bone-pelvis (9)	VMAT (2)
			Abemaciclib (3)		Ribs (4)	Tomotherapy (1)
					Skull (1)	
					Sternum (2)	
					Bone-extremities (1)	
Messer et al., 2019 [22]	Case report	1	Palbociclib	Fulvestrant	Supraclavicular node	3D-CRT
Chowdhary et al., 2019 [17]	Retrospective analysis	16	Palbociclib (16)	Fulvestrant (6)	Bone-spine (11)	3D-CRT (18)
				AI (10)	Bone-pelvis (4)	IMRT (2)
					Bone-extremities (3)	SBRT (2)
					Brain (4)	FSRS (1)
					Mediastinum (1)	
Beddock et al., 2020 [18]	Retrospective analysis	30	Palbociclib (30)	Fulvestrant (9)	Bone-spine (17)	3D-CRT (24)
				AI (21)	Bone-extremities (7)	IMRT (10)
					Choroidal (1)	SBRT (1)
					Brain (1)	
					Locoregional breast RT (9)	
Meattini et al., 2018 [27]	Case series	5	Ribociclib (5)	AI	Bone-spine (2)	3D-CRT (4)
					Bone-extremities (2)	VMAT (1)
					Bone-hip (1)	
Howlett et al., 2021 [31]	Letter to the editor	-	Palbociclib (28)	-	Bone-spine (23)	-
			Ribociclib (N/A)		Bone-others (16)	
			Abemaciclib (N/A)		Brain (2)	
Norman et al., 2022 [19]	Retrospective analysis	47	Palbociclib (47)	Fulvestrant (20)	Bone-pelvis (21)	3D-CRT (38)
				AI (27)	Bone-spine (12)	SBRT (5)
					Spine + pelvis (4)	IMRT (3)
					Bone-extremities (10)	Unknown (1)
Ratosa et al., 2020 [20]	Retrospective analysis	46	Palbociclib (30)	AI (14)	Bone-pelvis (11)	3D-CRT (41)
			Ribociclib (15)	TMX/LHRH (15)	Bone-spine (31)	IMRT (1)
			Abemaciclib (1)	Fulvestrant (3)	Bone-others (8)	Tomotherapy (2)
					Visceral/nodes mets (7)	SBRT (7)
					Brain (3)	2D-RT (11)
					Locoregional breast RT (2)	
Erjan et al., 2021 [23]	Case report	1	Ribociclib	AI (1)	Chest wall	SBRT
van Aken et al., 2021 [28]	Case series	3	Palbociclib	AI (1)	Bone-pelvis (3)	3D-CRT (3)
				Fulvestrant (2)	Mediastinal nodes	VMAT (1)
Dasgputa et al., 2021 [24]	Case report	1	Palbociclib	AI	Bone-pelvis and proximal femur	3D-CRT
Nasir et al., 2020 [25]	Case report	1	Palbociclib	AI	Bone-pelvis	3D-CRT
					Bone-spine (T10)	
Al-Rashdan et al., 2022 [21]	Retrospective analysis	132	Palbociclib (124)	AI (157)	Bone-pelvis (191)	3D-CRT (274)
			Ribociclib (8)	Fulvestrant/leuprolide (28)	Bone-other (61)	IMRT (18)
					Brain (20)	SBRT (28)
					Liver (1)	
					Lung (8)	
					Locorregional breast RT (39)	

AI = aromatase inhibitor; TMX = tamoxifen; IMRT = intensity-modulated radiation therapy; VMAT = volumetric modulated arc therapy; SBRT = stereotactic body radiation therapy; FSRS = fractionated stereotactic radiosurgery; 3D-CRT = three-dimensional conformal radiation therapy; 2D-RT = 2D radiation radiotherapy; N/A = not available.

**Table 2:** Most common toxicities among the reviewed published data.

MOST COMMON TOXICITIES	GRADE 2	GRADE 3	GRADE 4	CASES (N)	% (Number) of TOTAL POPULATION
NEUTROPENIA	14	48	3	65	43.9% (*N* = 148)
LEUKOPENIA	28	16	0	44	29.7% (*N* = 148)
ANEMIA	9	4	0	13	8.7% (*N* = 149)
THROMBOCYTOPENIA	4	2	0	6	4% (*N* = 150)
DERMATITIS	14	9	1	24	6.4% (*N* = 375)
DIARRHEA	15	3	2	20	5.8% (*N* = 345)
ESOPHAGITIS	11	4	0	15	4% (*N* = 375)
COLITIS	0	3	0	3	0.8% (*N* = 375)

**Table 3:** Grade 3 or higher nonhematological toxicities.

TOXICITY	GRADE	AUTHOR	CDKI4/6 AGENT	RT SITE	DELIVERED DOSE (Gy)	FRACTIONS (*N*)	TECHNIQUE
Dermatitis	G3	David S et al., 2021 [26]	Palbociclib	Right breast	36	12	N/A
		Messer et al., 2019 [22]	Palbociclib	Supraclavicular node	60	30	3D-CRT
		Beddock et al., 2020 [18]	Palbociclib	LR	50.4	28	N/A
		Howlett et al., 2021 [31]	NID	NID	NID	NID	NID
		Norman et al., 2022 [19]	Palbociclib	NID	NID	NID	NID
		Ratosa et al., 2020 [20]	NID	NID	NID	NID	NID
		Al-Rashdan et al., 2022 [21]	Palbociclib	Sternum	32	4	SBRT
Diarrhea	G3	Guerini et al., 2020 [16]	Palbociclib	Bone-pelvis	30	10	3D-CRT
		Ratosa et al., 2020 [20]	NID	NID	30	10	NID
		van Aken et al., 2021 [28]	Palbociclib	Bone-pelvis	20	5	N/A
Diarrhea	G4	Meattini et al., 2018 [27]	Ribociclib	Bone-hip	20	5	N/A
		Al-Rashdan et al., 2022 [21]	Ribociclib	Bone	20	5	3D-CRT
Colitis	G3	Kawamoto et al., 2019 [30]	Palbociclib	Bone-pelvis	30	10	3D-CRT
		van Aken et al., 2021 [28]	Palbociclib	Bone-pelvis	20	5	3D-CRT
		Dasgputa et al., 2021 [24]	Palbociclib	Bone-pelvis and proximal femur	30	10	3D-CRT
		Guerini et al., 2020 [16]	Palbociclib	Bone-pelvis	30	10	3D-CRT
Esophagitis	G3	Messer et al., 2019 [22]	Palbociclib	Supraclavicular node	60	30	3D-CRT
		Nasir et al., 2020 [25]	Palbociclib	Bone-spine	20	5	3D-CRT
		David S et al., 2021 [26]	Palbociclib	Bone-spine	30	10	N/A
			Palbociclib	Bone-spine	20	5	N/A
Pneumonitis	G5	David S et al., 2021 [26]	Palbociclib	Mediastinal node	20	5	N/A
